# Use of Point-of-Care Ultrasound for Early Identification of Acute Aortic Root Dissection

**DOI:** 10.1155/2022/7166230

**Published:** 2022-10-17

**Authors:** Kristina Thomas, Omar Amr

**Affiliations:** St. Joseph's Medical Center, Stockton, California, USA

## Abstract

Point-of-care ultrasound (POCUS) is becoming a frequently utilized imaging tool in the emergency department (ED) as it can aid in early diagnosis of many pathologies. This is a case report of a 55-year-old male who presented to the emergency department by ambulance for sudden onset chest pain followed by a syncopal episode. Point-of-care echocardiogram revealed a large pericardial effusion with a significantly dilated aortic root, concerning for aortic dissection. Patient was emergently taken for a computed tomography (CT) scan, which was only remarkable for an ascending thoracic aortic aneurysm but failed to show an aortic dissection flap. On repeat POCUS, a dissection intimal flap, large pericardial effusion with tamponade physiology, and aortic regurgitation were identified and later confirmed on transesophageal echocardiogram. This case report details a rare pathology that was correctly identified on initial POCUS before it was seen on CT scan.

## 1. Introduction

Thoracic aortic dissection is a rare but rapidly fatal pathology, with a mortality rate of 1-2% per hour after symptom onset [1]. The incidence is estimated at 3-4 cases per 100,000 persons per year [2]. It should be considered in all patients who present to the emergency department with acute onset of chest pain and rise higher on the differential in those with risk factors, such as male sex in the sixth decade of life and history of hypertension, atherosclerosis, or cardiac surgery [1]. Advanced imaging such as CT angiography is frequently relied upon, but is unrealistic for patients who are hemodynamically unstable and unable to be transported to the CT scanner. Therefore, POCUS is an alternative imaging technique that can aid in diagnosis of acute aortic dissection in the unstable patient [3].

This case report details a unique case of identifying a thoracic aortic dissection involving the aortic root on point-of-care transthoracic echocardiogram before the dissection flap was seen on CT angiography. This corroborates the importance of early and frequent POCUS to guide care in a critically ill patient and assist in rapid management and early specialist consultation to improve the patient's chances of survival.

## 2. Case Presentation

A 55-year-old male with no significant past medical history was presented to the emergency department by ambulance after experiencing sudden onset chest pain followed by a syncopal episode. The chest pain was located substernally and radiated through the chest to the midscapular area. Associated symptoms included shortness of breath, lightheadedness, nausea, and diaphoresis. Upon arrival to the emergency department, the patient was alert and oriented, continuing to complain of chest pain radiating to the back. Initial vitals were remarkable for tachycardia and hypoxia, requiring 15 liters of oxygen via a non-rebreather, though the patient was normotensive at time of arrival. On physical exam, the patient appeared uncomfortable, diaphoretic, and had labored respirations. The initial electrocardiogram (ECG) was remarkable for sinus tachycardia with ST depressions in the anterolateral leads, and negative for ST segment elevation myocardial infarction (STEMI).

**Figure 1:**
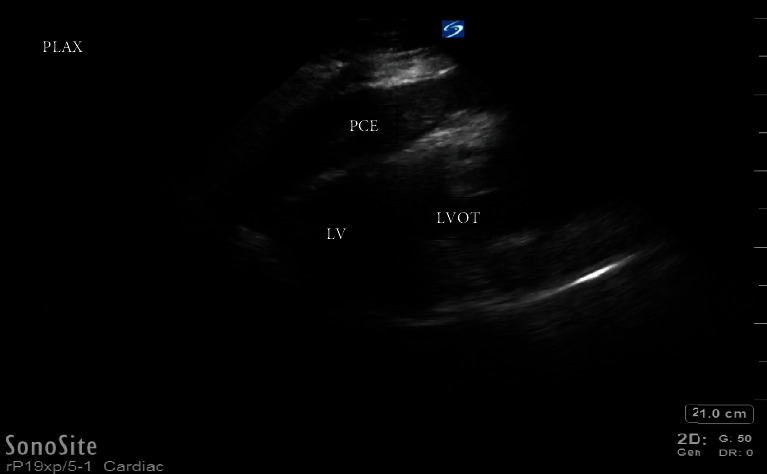
Parasternal long axis view of the heart displaying a pericardial effusion with dilated aortic root, concerning for aortic root dissection. LV: left ventricle; LVOT: left ventricular outflow tract; PCE: pericardial effusion.

**Figure 2:**
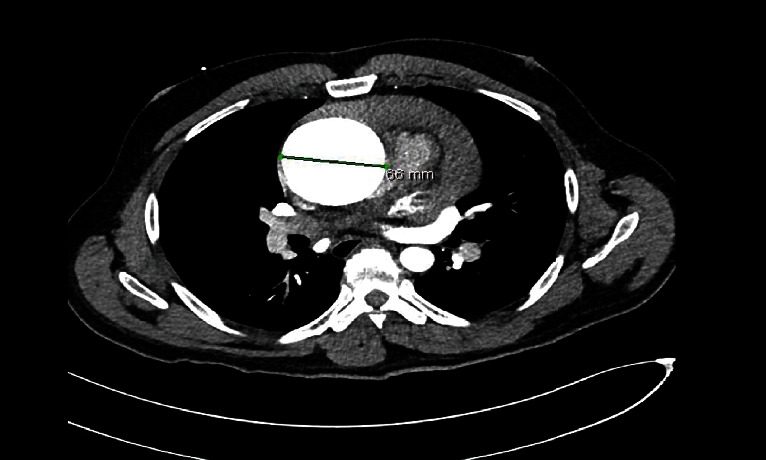
CT imaging with 6.6 cm aneurysm of the ascending thoracic aorta without dissection. Large pericardial effusion.

**Figure 3:**
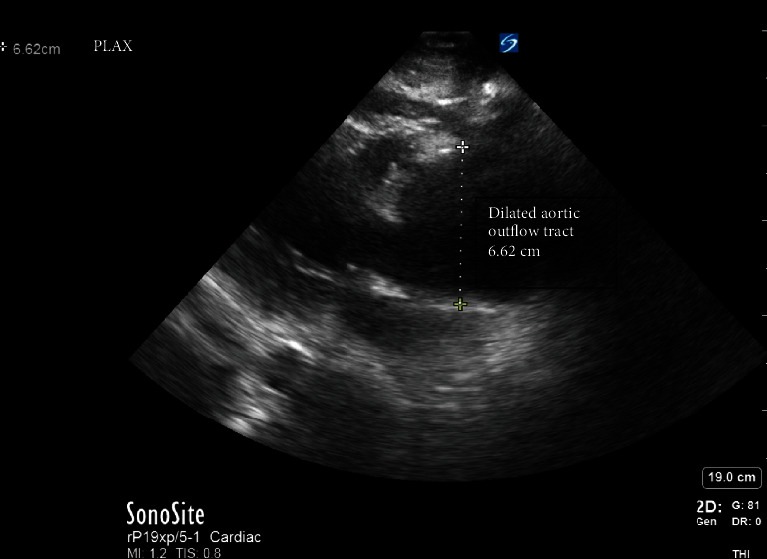
Parasternal long axis view of the heart, demonstrating a dilated aortic outflow tract measuring 6.62 cm during diastole. Normal size of the aortic outflow tract is less than 4 cm.

**Figure 4:**
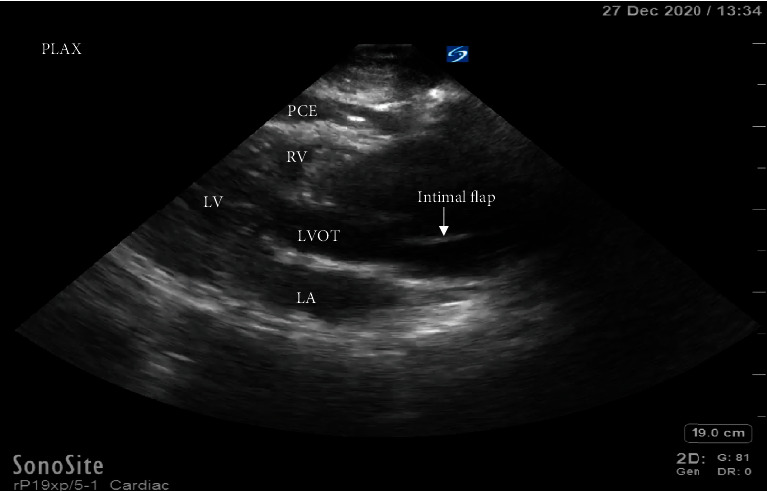
Parasternal long axis displaying the intimal flap within the aortic outflow tract. LA: left atrium; LV: left ventricle; RV: right ventricle; PCE: pericardial effusion; LVOT: left ventricular outflow tract.

**Figure 5:**
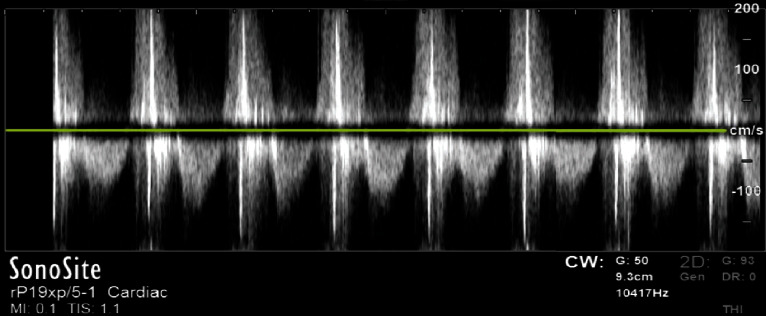
Velocity time integral (VTI) demonstrating aortic regurgitation. The negative deflections show the normal forward flow of blood through the aortic valve, but the presence of positive deflections show the regurgitant flow.

**Figure 6:**
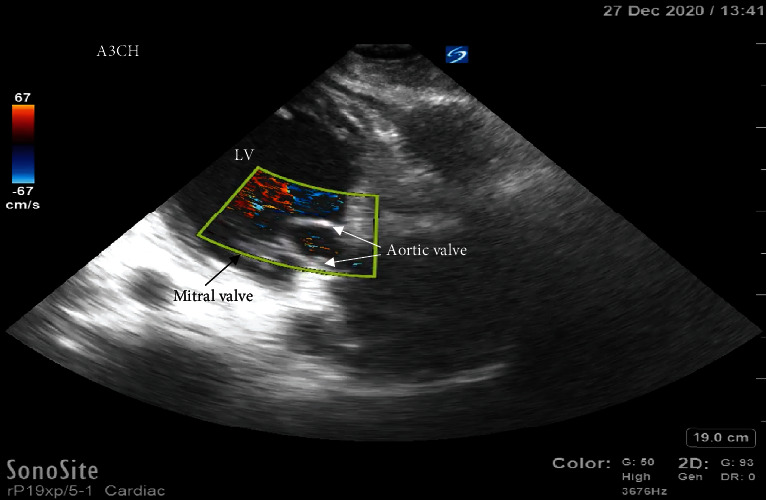
Color flow Doppler demonstrating regurgitant flow through the aortic valve, which has retracted back into the left ventricle because of the aortic root dissection.

A point-of-care transthoracic echocardiogram was immediately performed. This revealed a pericardial effusion and dilated aortic root (Figure 1). In clinical context, this was concerning for a thoracic aortic dissection. The patient was stable enough for CT scan at this time, which also demonstrated a pericardial effusion and a 6.6-centimeter ascending thoracic aortic aneurysm but no CT evidence of aortic dissection (Figure 2). After returning to the ED from CT scan, point-of-care transthoracic echocardiogram was repeated, which showed a larger pericardial effusion with tamponade physiology, dilated aortic root measuring 6.62-centimeter (Figure 3), an intimal flap within the left ventricular outflow tract (Figure 4), aortic regurgitation demonstrated on velocity time integrals (Figure 5), and color flow Doppler (Figure 6).

Due to the above findings on the POCUS, cardiothoracic surgery (CT surgery) was consulted and requested to come emergently to the bedside. When the patient was brought back to the ED from CT scan, he quickly became more obtunded and hypotensive. Two central venous catheters were placed and he was emergently intubated for airway protection using rapid sequence intubation with etomidate and rocuronium. A massive transfusion protocol was initiated. Shortly, after intubation, the patient went into pulseless electrical activity cardiac arrest and ACLS protocol was initiated. A repeat point-of-care echocardiogram was obtained, which again showed the large pericardial effusion, now with tamponade physiology, and an intimal flap was noted in the left ventricular outflow tract on the parasternal long view. A pericardiocentesis under ultrasound guidance was performed which evacuated approximately 50 cc of bright red blood from the pericardial sac. Return of spontaneous circulation was subsequently achieved.

The patient was then taken to the operating room for CT surgery. Prior to the beginning of the operation, the patient went into a pulseless electrical activity cardiac arrest. Transesophageal echocardiography performed by the anesthesiologist revealed a trivial pericardial effusion without cardiac activity, and a large dissection involving the aortic root, which occluded the right and left main coronary arteries. Due to the poor prognosis, resuscitative measures were stopped and the patient was declared deceased.

## 3. Discussion

Acute thoracic aortic dissection is a rare condition with an annual incidence of 3 per 100,000 individuals [4]. The mortality rate ranges from 18 to 30% [4, 5]. The Stanford classification system is widely accepted and differentiates types of dissection by location; a Stanford type A dissection involves the ascending aorta, whereas a Stanford type B type involves the descending aorta [6]. The diagnosis is classically made with CT angiography to identify the dissection flap. This modality has a sensitivity of 95% and a specificity of 98% for acute thoracic aortic dissection [6]. Because this diagnosis needs to be made quickly, especially in unstable patients, point-of-care transthoracic echocardiography (TTE) is a rapid and noninvasive imaging study that should be used to aid in the diagnosis of acute aortic dissection. Particularly useful images include a parasternal long axis and apical views, as these images can reveal pericardial effusion, aortic root dilatation, aortic regurgitation, or an intimal aortic flap [3]. Transthoracic echocardiography carries a sensitivity of 78-90% and a specificity of 87-96% for diagnosing thoracic aortic dissection, with more recent data showing higher sensitivity of 97% and specificity of 99-100% in patients with optimal windows [7, 8]. If an intimal flap is identified, bedside echocardiogram has a sensitivity of 67-80% and specificity of 99-100% [9].

To our knowledge, this is the first case report to date where a diagnosis of a thoracic aortic dissection involving the aortic root was identified on point-of-care echocardiogram, but not on CT angiography. In this unique case, the likely reason for identifying the aortic root dissection on POCUS rather than CT angiography is due to an active aortic dissection starting at the aortic root, with subsequent retrograde extension into the ascending aorta, which seemed to occur after CT imaging. When the patient decompensated after CT scan, the repeat point-of-care echocardiogram demonstrated direct findings of an acute aortic root dissection. An apical three-chamber view was obtained, which showed the intimal flap within the severely dilated aortic outflow tract. Pulsed wave Doppler was used over the left ventricular outflow tract to calculate the velocity time integral (VTI); this demonstrated positive deflections which correlated with regurgitant flow through the aortic valve. The aortic valve was also visualized in a retracted position within the left ventricle due to the loss of annular insertion on the left ventricle from the dissection.

Once in the operating room, the patient underwent a TEE, which demonstrated a 7-centimeter ascending aortic aneurysm and an aortic root dissection, which appeared to occlude the main coronary arteries. The paradigm of the TEE is identifying the intimal flap, which carries a sensitivity and specificity of greater than 95% [10]. This is a particularly useful study in unstable patients and can be used bedside in the ED on intubated patients, as well as in the operating room.

The peculiar nature of this case involves the progression of the dissection throughout the case; this explains the evolution of findings on the imaging studies. The first TTE and CT angiography only showed the large pericardial effusion because the dissection was likely in progress and had not yet extended into the ascending aorta; only the aortic root, and possibly the coronary arteries were involved, at this point leading to the pericardial effusion. When the second TTE was performed after CT imaging, the patient began to decompensate which corroborates the extension of the dissection to involve the ascending aorta; this explains why the intimal flap, aortic regurgitation, and retracted aortic valve were seen only on repeat TTE. This enlarging dissection therefore led to hemodynamic instability and ultimately cardiac arrest. If the CT imaging were repeated after the change in hemodynamics, it is expected that the aortic dissection would be seen at that time.

While this patient unfortunately expired in the operating room, the case highlights the importance of POCUS in the Emergency Department for diagnosis of fatal pathologies, as it is rapid, portable, noninvasive, and repeatable. We acknowledge that while POCUS should not replace advanced imaging that aids the cardiothoracic surgeon in operation planning, it can streamline management and early consultation with specialists to decrease morbidity and mortality.

## Data Availability

All data are available within the manuscript.
